# Common *NFKBIL2 *polymorphisms and susceptibility to pneumococcal disease: a genetic association study

**DOI:** 10.1186/cc9377

**Published:** 2010-12-20

**Authors:** Stephen J Chapman, Chiea C Khor, Fredrik O Vannberg, Anna Rautanen, Andrew Walley, Shelley Segal, Catrin E Moore, Robert JO Davies, Nicholas P Day, Norbert Peshu, Derrick W Crook, James A Berkley, Thomas N Williams, J Anthony Scott, Adrian VS Hill

**Affiliations:** 1 The Wellcome Trust Centre for Human Genetics, University of Oxford, Roosevelt Drive, Oxford OX3 7BN, UK; 2 Oxford Centre for Respiratory Medicine, Churchill Hospital Site, Oxford Radcliffe Hospitals, Roosevelt Drive, Oxford OX3 7LJ, UK; 3 NIHR Oxford Biomedical Research Centre, Respiratory Medicine, John Radcliffe Hospital, Headley Way, Oxford OX3 9DU, UK; 4 Current address: Division for Infectious Diseases, Genome Institute of Singapore, 60 Biopolis Street, Singapore; 5 Current address: Section of Genomic Medicine, Imperial College London, Hammersmith Hospital, Du Cane Road, London W12 0NN, UK; 6 Department of Paediatrics, John Radcliffe Hospital, Headley Way, Oxford OX3 9DU, UK; 7 Centre for Clinical Vaccinology and Tropical Medicine, Churchill Hospital, Roosevelt Drive, Oxford OX3 7LJ, UK; 8 Kenya Medical Research Institute/Wellcome Trust Programme, Centre for Geographic Medicine Research, Coast, Kilifi District Hospital, P.O. Box 230-80108, Kilifi, Kenya; 9 Department of Microbiology, John Radcliffe Hospital, Headley Way, Oxford OX3 9DU, UK; 10 Nuffield Department of Clinical Medicine, John Radcliffe Hospital, Headley Way, Oxford OX3 9DU, UK; 11 INDEPTH Network, 11 Mensah Wood Street, East Legon, P. O. Box KD 213, Kanda, Accra, Ghana

## Abstract

**Introduction:**

*Streptococcus pneumoniae *remains a major global health problem and a leading cause of death in children worldwide. The factors that influence development of pneumococcal sepsis remain poorly understood, although increasing evidence points towards a role for genetic variation in the host's immune response. Recent insights from the study of animal models, rare human primary immunodeficiency states, and population-based genetic epidemiology have focused attention on the role of the proinflammatory transcription factor NF-κB in pneumococcal disease pathogenesis. The possible role of genetic variation in the atypical NF-κB inhibitor IκB-R, encoded by *NFKBIL2*, in susceptibility to invasive pneumococcal disease has not, to our knowledge, previously been reported upon.

**Methods:**

An association study was performed examining the frequencies of nine common *NFKBIL2 *polymorphisms in two invasive pneumococcal disease case-control groups: European individuals from hospitals in Oxfordshire, UK (275 patients and 733 controls), and African individuals from Kilifi District Hospital, Kenya (687 patients with bacteraemia, of which 173 patients had pneumococcal disease, together with 550 controls).

**Results:**

Five polymorphisms significantly associated with invasive pneumococcal disease susceptibility in the European study, of which two polymorphisms also associated with disease in African individuals. Heterozygosity at these loci was associated with protection from invasive pneumococcal disease (rs760477, Mantel-Haenszel 2 × 2 χ^2 ^= 11.797, *P *= 0.0006, odds ratio = 0.67, 95% confidence interval = 0.53 to 0.84; rs4925858, Mantel-Haenszel 2 × 2 χ^2 ^= 9.104, *P *= 0.003, odds ratio = 0.70, 95% confidence interval = 0.55 to 0.88). Linkage disequilibrium was more extensive in European individuals than in Kenyans.

**Conclusions:**

Common *NFKBIL2 *polymorphisms are associated with susceptibility to invasive pneumococcal disease in European and African populations. These findings further highlight the importance of control of NF-κB in host defence against pneumococcal disease.

## Introduction

Respiratory infection is the single largest contributor to the global burden of disease and the leading cause of death in children worldwide [1,2]. *Streptococcus pneumoniae *(the pneumococcus) remains the most common cause of community-acquired pneumonia in Europe and the United States [3]. In addition to pneumonia, pneumococcal infection may also manifest as invasive disease, defined by the isolation of *S. pneumoniae *from a normally sterile site such as blood (bacteraemia) or cerebrospinal fluid (meningitis). Although asymptomatic colonisation of the nasopharynx by the pneumococcus is widespread in the population, invasive pneumococcal disease (IPD) occurs in only a minority of individuals [4,5]. The factors that influence development of invasive disease remain poorly understood, although increasing evidence points towards a role for genetic variation in the host's immune response [5]. In particular, recent insights from the study of animal models, rare human primary immunodeficiency states, and population-based genetic epidemiology have focused attention on the control of the proinflammatory transcription factor NF-κB in the development of IPD [5-10].

NF-κB plays a key regulatory role in a diverse array of cellular processes, including innate and adaptive immune responses [11,12]. In unstimulated cells, NF-κB transcription factor subunits are prevented from binding DNA through associations with the inhibitor of NF-κB (IκB) protein family. Stimulation of a variety of immune receptors (including Toll-like receptors, T-cell and B-cell antigen receptors and members of the IL-1 and TNF receptor superfamilies) leads to phosphorylation and degradation of the IκB inhibitors and release of NF-κB, which induces transcription of proinflammatory target genes [11,12]. Genes that are activated by NF-κB include those encoding cytokines (for example, IL-1, IL-2, IL-6, TNFα) and chemokines (for example, IL-8, RANTES), as well as acute phase response proteins, adhesion molecules, antimicrobial peptides and inducible enzymes [11,12].

Members of the IκB family are characterised by multiple ankyrin repeats and can be subdivided into the so-called classical IκBs (IκB-α, IκB-β, IκB-ε), unusual IκBs (IκB-R, IκB-ζ, IκB-L, Bcl-3), and NF-κB precursors [12,13]. Of these, the least well studied is IκB-R (IκB-related), encoded by the gene *NFKBIL2. *The gene was first cloned in 1995 from human lung alveolar epithelial cells, and a modified sequence was published in 2000 [14,15]. The gene contains only three ankyrin-repeat motifs, fewer than other IκB members, and its exons have a more complicated structure than that seen in other IκBs; overall there is only weak homology between IκB-R and other IκB proteins, leading to the suggestion that IκB-R may in fact not be a member of the IκB family [15]. There is evidence, however, to support an interaction of IκB-R with NF-κB. IκB-R was first shown to inhibit DNA binding by NF-κB in electrophoretic mobility shift assays [14], and overexpression of *NFKBIL2 *in lung alveolar epithelial cells was subsequently reported to significantly upregulate the production of RANTES (now renamed chemokine C-C motif ligand 5 (CCL5)) protein following stimulation with TNFα or IL-1α, although it had no effect on other NF-κB-responsive chemokines such as IL-8 [16].

Increasing evidence supports a central role for the control of NF-κB in susceptibility to severe infectious disease in humans. A mutation in the gene *NFKBIA *encoding the classical inhibitor IκB-α has been described in two patients with primary immunodeficiency [8]. In addition, population-based case-control studies of IPD have reported associations with polymorphisms in the IκB-encoding genes *NFKBIA *and *NFKBIZ *[9,10]. These findings raise the possibility that variation in additional IκBs such as IκB-R may also contribute to IPD susceptibility. No functional or disease-associated polymorphisms have previously been reported in *NFKBIL2*, however. To investigate this further we studied the frequencies of *NFKBIL2 *polymorphisms in individuals with IPD and healthy controls of both European and African descent.

## Materials and methods

### Sample information

The UK Caucasian IPD sample collection has been previously described [17]. Blood samples were collected on diagnosis from all hospitalised patients with microbiologically-proven IPD (defined by the isolation of *S. pneumoniae *from a normally sterile site, most commonly blood) as part of an enhanced active surveillance programme between June 1995 and May 2001 in three hospitals in Oxfordshire, UK: John Radcliffe Hospital, Horton General Hospital, and Wycombe General Hospital. There were no exclusion criteria for the study. DNA samples were available for study from 275 patients. Clinical details, including age, gender, clinical presentation and the presence of underlying risk factors, were recorded. During the study, Oxfordshire was a region of very low HIV prevalence and HIV testing was not routinely performed. Frequencies of initial clinical presentation were as follows: pneumonia 69%, isolated bacteraemia 15%, meningitis 11%, and other presentations 5%. The mean age of the patients was 58 years, ranging from 0 to 94 years; 50% were male. Pneumococcal serotypes were identified using polyclonal rabbit antisera (Statens Seruminstitut, Copenhagen, Denmark). The distribution of serotypes was very similar to that of previous UK studies, with serotype 14 being the commonest.

The control group comprised a combination of 163 UK healthy adult blood donors and 570 cord blood samples. For the cord samples, blood was collected anonymously from the discarded umbilical cords of healthy neonates born at the John Radcliffe Hospital, Oxford, UK, as previously described [17]. Examination of microsatellite markers excluded contamination with maternal DNA. The use of DNA from cord blood samples is intended to reveal background population allele frequencies; recent large-scale genotyping of a UK birth cohort control group for association studies of multiple disease phenotypes has confirmed the validity of such an approach [18]. The mean age of the adult blood donors was 38 years, and 50% were male; 54% of the cord blood donors were male. Individuals of non-European ancestry were excluded from cases and controls. The study was approved by the Oxford Local Research Ethics Committee and informed consent was obtained from all participants.

The Kenyan bacteraemia case-control collection has also been previously described [19]. Kenyan children (<13 years old) with bacteraemia were recruited from Kilifi District hospital between 1998 and 2002. The 687 bacteraemic cases comprised patients with isolated Gram-positive and Gram-negative infections, diagnosed using standard blood culture techniques. The most frequent organisms isolated were *S. pneumoniae *(25%), non-Typhi Salmonella species (16%), *Haemophilus influenzae *(14%), and *Escherichia coli *(8%), as well as other less common bacteria. The 550 community controls were individually matched to a subset of the cases on the basis of time (recruited within 14 days), location of homestead, age, and sex. Only children with complete data for HIV, malnutrition, and malaria status were included in the analysis. Ethical approval for the study was given by the Kenya Medical Research Institute National Scientific Steering and Research Committees and informed consent was obtained from all participants.

### Genotyping techniques

DNA extraction from blood was performed using Nucleon II kits (Scotlab Bioscience, Buckingham, UK). Polymorphisms within *NFKBIL2 *were selected from the dbSNP and ensembl databases on the basis of their probable functionality [20,21], as well as to provide an overview of linkage disequilibrium (LD) across the gene and flanking regions. Genotyping was performed using the Sequenom Mass-Array^® ^MALDI-TOF primer extension assay [22]; primer sequences are listed in Table 1. A touch-down PCR protocol was used, with cycling conditions as follows: 95ºC for 15 minutes; 94ºC for 20 seconds; 65ºC for 30 seconds; 72ºC for 30 seconds; steps 2 to 4 repeated for five cycles; 94ºC for 20 seconds; 58ºC for 30 seconds; 72ºC for 30 seconds; steps 5 to 7 repeated for five cycles; 94ºC for 20 seconds; 53ºC for 30 seconds; 72ºC for 30 seconds; steps 8 to 10 repeated for 38 cycles; and final extension at 72ºC for 3 minutes. Each genotyping plate contained a mixture of case and control samples.

**Table 1:** Primer sequences for *NFKBIL2 *polymorphism genotyping using the Sequenom Mass-Array^® ^MALDI-TOF primer extension assay

Polymorphism	PCR primer sequences	Extension primer sequences
rs10448143	ACGTTGGATGGGAACTGGAGCACGGGCTT	CACGGGCTTCCCGTGGC
	ACGTTGGATGAAGATGTCTCAGGGTCTTGG	
rs2170096	ACGTTGGATGACTCCCAACCTCAGGTCATC	GCTGGGATCACAGGCGTGAG
	ACGTTGGATGAGAAATTGGGTTGTCAGCCG	
rs4925858	ACGTTGGATGTGCAGGAGGCAGGAAATCCA	GCAGGCCTGGGTGTGAG
	ACGTTGGATGATGCTTTGGATGGGCAAGGG	
rs760477	ACGTTGGATGAAAGGGAGGGCTCCAGAAGAC	TCCAGAAGACGGGATTGCCCAA
	ACGTTGGATGGCGTTTTCTGCCTCCTGAAC	
rs2306384	ACGTTGGATGGGAAATGCAAGGTGCCGCTG	TGCCGCTGGCCCTCACCGC
	ACGTTGGATGAGCCACAGCGGAGAGCGAAG	
rs4082353	ACGTTGGATGTAGTCTGCTCTGAAGGTTGG	TGGAGAGACCAGAGGCAGA
	ACGTTGGATGTGATCCCAGCTCCTAAAACC	
rs2272658	ACGTTGGATGAACTGTTCCTGAGGCACTCC	GAGGCACTCCAGGATGGAGC
	ACGTTGGATGTAGAGCCCAGAGTGCTACCC	
rs13258200	ACGTTGGATGAAAGTGACTGGCAGCTTCTG	CCTCCTAGGGCTCTGAGTTCCTGC
	ACGTTGGATGTGGTGGTGTTGGTGTAGTTG	
rs4380978	ACGTTGGATGCAAAGCCTTCCAGTTTGGAC	AGATGAAACGGGTGCCCC
	ACGTTGGATGCTGCACACACTCACCATAAG	

General PCR conditions for amplifying products prior to sequencing were as follows: 95ºC for 15 minutes, and then 40 cycles of 95ºC for 30 seconds, 55 to 65ºC for 30 seconds, and 72ºC for 60 seconds, followed by 72ºC for 5 minutes. Direct sequencing was performed using BigDye v3.1 terminator mix (Applied Biosystems, Foster City, CA, USA) followed by ethanol precipitation. Plates were run on an ABI 3700 capillary sequencer and sequence analysis was performed with the Lasergene DNAstar package using SeqMan software (DNASTAR Inc., Madison, WI, USA). Primer sequences are listed in Table 2.

**Table 2:** Primer sequences used for direct sequencing

Name	Forward primer sequence	Reverse primer sequence
NFKBIL2_prom	CGTCAGTCTATCTGGACAC	CTCGCGCTCCAGGCTCATGCTC
NFKBIL2_ex1_2	GAGCATGAGCCTGGAGCGCGAG	CAAGGCTGCGTCAGGTCAGGTG
NFKBIL2_int2	CACCTGACCTGACGCAGCCTTG	CAGTGGCTTCACGCTGTATGCAGC
NFKBIL2_ex3	GCTGCATACAGCGTGAAGCCACTG	GGATAAAGAGCTGACGATCTCCAG
NFKBIL2_ex4	CTGGAGATCGTCAGCTCTTTATCC	TACTTCCTCCAGGAACAAG
NFKBIL2_ex5_6	CTTGTTCCTGGAGGAAGTA	GAGAGCCCTGTACACACCTG

### Statistical analysis

Statistical analysis of genotype associations and logistic regression was performed using the program SPSS v16.0 (SPSS, Inc., Chicago, IL, USA). Two-tailed tests of significance were used for all analysis. Uncorrected *P *values are presented throughout; appropriate significance thresholds in the setting of multiple testing are described in the Discussion. Tarone's homogeneity of odds ratio (OR) testing was performed to compare ORs between study groups; if appropriate, study groups were combined and stratified using Mantel-Haenszel testing (SPSS v16.0). Analysis of LD was performed using the Haploview v4.1 program [23]. Haplotype blocks were defined as regions demonstrating strong evidence of historical recombination between <5% of SNP-pair comparisons [24]. All control genotype distributions were in Hardy-Weinberg equilibrium.

## Results

The initial genotyping approach utilised the UK Caucasian IPD case-control study group and focused on three SNPs within *NFKBIL2*: rs760477, rs2306384, and rs4082353. Whilst both rs760477 and rs4082353 are intronic, rs2306384 encodes a serine/glycine substitution at position 334 of the IκB-R protein. Each of these SNPs was found to be common in Europeans (minor allele frequencies approaching 50%) and to associate with susceptibility to IPD (*P *= 0.002 to 0.007; Table 3). Logistic regression analysis demonstrated no effect of age, comorbidity or gender on genotype. Genotyping was then extended to flanking SNPs in both directions spanning a 74 kb region across chromosome 8q24.3 to delineate the extent of LD and disease association. Twelve SNPs were found to be either nonpolymorphic or extremely rare (minor allele frequency <0.01) and could not be analysed further (Table 3). A further six SNPs were polymorphic, of which two associated with IPD susceptibility at the 0.05 significance level and one trended towards association (*P *= 0.035 to 0.085; Table 3). In each case the minor alleles were again found to be common, and the direction of association was one of heterozygote protection against IPD (Table 3).

**Table 3:** *NFKBIL2 *and flanking gene polymorphism genotype frequencies in European individuals with IPD and controls

Polymorphism/location^a ^(major/minor allele)	Status	Genotype distribution^b^			Genotypic 3 × 2 chi-square (*P *value)	Heterozygote protection model^c^	
				
		AA	Aa	aa		OR (95% CI)	*P *value^d^
rs10448143, -5,224, 5' upstream (C/T)	Control	203 (57.7%)	126 (35.8%)	23 (6.5%)	1.245 (0.537)	1.17 (0.79 to 1.72)	0.425
	IPD	88 (56.1%)	62 (39.5%)	7 (4.5%)			
rs2170096, -4,368, 5' upstream (C/G)	Control	164 (24.3%)	349 (51.7%)	162 (24.0%)	4.927 (0.085)	0.71 (0.51 to 0.99)	**0.036**
	IPD	53 (26.4%)	87 (43.3%)	61 (30.3%)			
rs4925858, -3,771, 5' upstream (G/A)	Control	185 (27.1%)	360 (52.7%)	138 (20.2%)	6.664 (**0.036**)	0.69 (0.50 to 0.95)	**0.016**
	IPD	66 (29.6%)	97 (43.5%)	60 (26.9%)			
rs760477, -263, *NFKBIL2 *intron 4 (C/T)	Control	188 (26.3%)	370 (51.7%)	158 (22.1%)	9.810 (**0.007**)	0.64 (0.48 to 0.85)	**0.002**
	IPD	82 (31.3%)	106 (40.5%)	74 (28.2%)			
rs2306384, +2,754, Ser/Gly, *NFKBIL2 *exon 11 (A/G)	Control	158 (24.6%)	336 (52.4%)	147 (22.9%)	12.329 (**0.002**)	0.61 (0.45 to 0.83)	**0.001**
	IPD	67 (27.0%)	100 (40.3%)	81 (32.7%)			
rs4082353, +12,589, *NFKBIL2 *intron 25 (G/T)	Control	175 (27.2%)	327 (50.8%)	142 (22.0%)	9.932 (**0.007**)	0.66 (0.49 to 0.89)	**0.005**
	IPD	70 (28.6%)	99 (40.4%)	76 (31.0%)			
rs2272658, +16,899, *VPS28 *intron 4 (C/T)	Control	156 (24.6%)	330 (52.1%)	147 (23.2%)	6.684 (**0.035**)	0.68 (0.49 to 0.95)	**0.023**
	IPD	47 (25.7%)	78 (42.6%)	58 (31.7%)			
rs13258200, +36,964, *CPSF1 *intron 2 (A/C)	Control	258 (39.2%)	317 (48.1%)	84 (12.7%)	0.364 (0.833)	0.95 (0.72 to 1.26)	0.739
	IPD	107 (38.9%)	129 (46.9%)	39 (14.2%)			
rs4380978, +68,695, *ADCK5 *intron 1 (G/C)	Control	212 (31.7%)	347 (51.9%)	110 (16.4%)	0.227 (0.893)	0.94 (0.70 to 1.26)	0.696
	IPD	78 (32.0%)	123 (50.4%)	43 (17.6%)			

*VPS28*, vacuolar protein sorting 28; *CPSF1*, cleavage and polyadenylation-specific factor 1; *ADCK5*, aarF domain-containing kinase 5; OR, odds ratio; CI, confidence interval; IPD, invasive pneumococcal disease. The following *NFKBIL2 *SNPs were nonpolymorphic or very rare (minor allele frequency <0.01) in the UK Caucasian population studied: rs7459910; rs2306383; rs4448319; rs3802163; rs4925856; rs2242264; rs2620660; rs741970; rs4925857; rs6985339; rs2928378; rs12677973. ^a^SNP positions listed are relative to the start of translation (in exon 5). ^b^Number of individuals (%). ^c^Comparison of heterozygotes [Aa] with homozygotes [AA + aa]. ^d^2 × 2 chi-squared comparison, one degree of freedom. *P *values below 0.05 are highlighted in bold.

**Figure 1:**
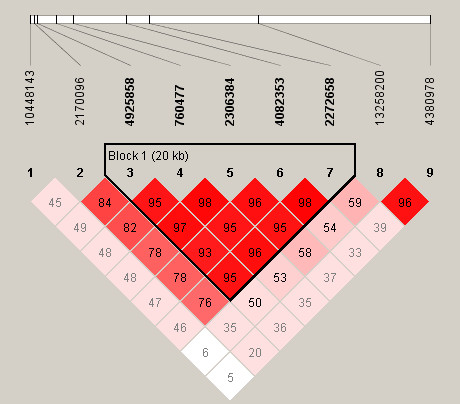
**Relative position of SNPs and linkage disequilibrium map for *NFKBIL2 *in the UK populations studied**. Polymorphisms are identified by their dbSNP rs numbers, and their relative positions are marked by vertical lines within the white horizontal bar. Numbers within squares indicate the *D*' value expressed as a percentile. Red squares indicate pairs in strong linkage disequilibrium (LD) with LOD scores for LD ≥2, pink squares *D*' <1 with LOD ≥2, and white squares *D*' <1.0 and LOD <2.

**Figure 2:**
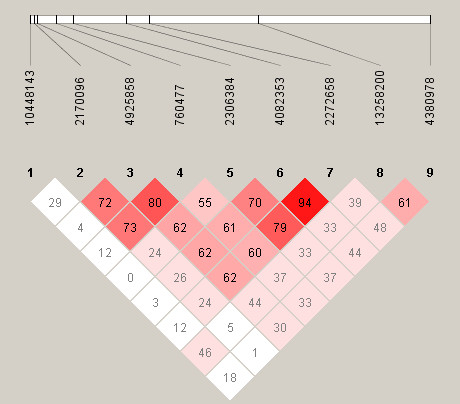
**Relative position of SNPs and linkage disequilibrium map for *NFKBIL2 *in the Kenyan populations studied**. Polymorphisms are identified by their dbSNP rs numbers, and their relative positions are marked by vertical lines within the white horizontal bar. Numbers within squares indicate the *D*' value expressed as a percentile. Red squares indicate pairs in strong linkage disequilibrium (LD) with LOD scores for LD ≥2, pink squares *D*' <1 with LOD ≥2, and white squares *D*' <1.0 and LOD <2.

The extent of LD between SNPs was next assessed. All five IPD-associated SNPs were found to be located within a 20 kb block of strong LD in this European population (Figure 1). The absence of association with SNPs outside this block suggests that the causative locus is indeed localised to this 20 kb region, which contains the entire *NFKBIL2 *gene as well as the neighbouring gene in a 3' direction, vacuolar protein sorting 28 (*VPS28*). This extensive LD presents a considerable challenge, however, in identifying the IPD-causative polymorphism. The extent of LD in African populations is typically shorter than in Europeans [24], and this can be advantageous when attempting to fine map an extensive region of disease association. With this in mind, all nine polymorphisms were then genotyped in the Kenyan bacteraemia case-control study (Tables 4 and 5). The LD was noted to be much less extensive in this African population, and no haplotype blocks were predicted by the Gabriel algorithm within the region studied (Figure 2).

**Table 4:** *NFKBIL2 *and flanking gene polymorphism allele frequencies: European IPD and African bacteraemia case-control studies

Polymorphism/location	UK Caucasian study		Kenyan study	
		
	Minor allele frequency (%)	*P *value^a^	Minor allele frequency (%)	*P *value^a^
rs10448143, -5,224	24.3	0.537	4.7	0.250^b^
				
rs2170096, -4,368	49.6	0.085	38.2	0.145
				
rs4925858, -3,771	47.1	**0.036**	30.7	0.128
				
rs760477, -263	48.1	**0.007**	25.7	**0.009**
				
rs2306384, +2,754	49.8	**0.002**	33.5	0.427
				
rs4082353, +12,589	48.5	**0.007**	33.4	0.857
				
rs2272658, +16,899	49.9	**0.035**	36.7	0.927
				
rs13258200, +36,964	37.0	0.833	40.2	0.801
				
rs4380978, +68,695	42.5	0.893	32.6	0.648
				

^a^*P *values are derived from 3 × 2 chi-squared comparisons of genotypes (two degrees of freedom). ^b^Fisher's exact test (two-tailed). *P *values below 0.05 are highlighted in bold.

**Table 5:** *NFKBIL2 *and flanking gene polymorphism genotype frequencies in Kenyan individuals with bacteraemia and controls

Polymorphism/location^a ^(major/minor allele)	Status	Genotype distribution^b^			Genotypic 3 × 2 chi-square (*P *value)	Heterozygote protection model^c^	
				
		AA	Aa	aa		OR (95% CI)	*P *value^d^
rs10448143, -5,224, 5' upstream (C/T)	Control	433 (89.6%)	50 (10.4%)	0 (0%)	0.250^e^	0.78 (0.52 to 1.17)	0.226
	Bacteraemia	598 (91.4%)	54 (8.3%)	2 (0.3%)			
rs2170096, -4,368, 5' upstream (C/G)	Control	128 (38.7%)	136 (41.1%)	67 (20.2%)	0.145	0.93 (0.66 to 1.31)	0.672
	Bacteraemia	107 (45.7%)	92 (39.3%)	35 (15.0%)			
rs4925858, -3,771, 5' upstream (G/A)	Control	255 (46.4%)	247 (44.9%)	48 (8.7%)	0.128	0.80 (0.64 to 1.00)	0.053
	Bacteraemia	343 (49.9%)	271 (39.4%)	73 (10.6%)			
rs760477, -263, *NFKBIL2 *intron 4 (C/T)	Control	262 (53.0%)	192 (38.9%)	40 (8.1%)	**0.009**	0.68 (0.53 to 0.87)	**0.002**
	Bacteraemia	403 (60.6%)	201 (30.2%)	61 (9.2%)			
rs2306384, +2,754, Ser/Gly, *NFKBIL2 *exon 11 (A/G)	Control	238 (45.0%)	224 (42.3%)	67 (12.7%)	0.427	0.84 (0.65 to 1.09)	0.195
	Bacteraemia	214 (47.9%)	171 (38.3%)	62 (13.8%)			
rs4082353, +12,589, *NFKBIL2 *intron 25 (G/T)	Control	160 (47.6%)	132 (39.3%)	44 (13.1%)	0.857	1.05 (0.75 to 1.47)	0.785
	Bacteraemia	109 (45.4%)	97 (40.4%)	34 (14.2%)			
rs2272658, +16,899, *VPS28 *intron 4 (C/T)	Control	135 (40.9%)	145 (43.9%)	50 (15.2%)	0.927	0.96 (0.69 to 1.33)	0.791
	Bacteraemia	113 (42.5%)	114 (42.9%)	39 (14.7%)			
rs13258200, +36,964, *CPSF1 *intron 2 (A/C)	Control	212 (37.7%)	245 (43.6%)	105 (18.7%)	0.801	1.06 (0.85 to 1.33)	0.601
	Bacteraemia	259 (37.5%)	311 (45.1%)	120 (17.4%)			
rs4380978, +68,695, *ADCK5 *intron 1 (G/C)	Control	246 (45.6%)	231 (42.8%)	63 (11.6%)	0.648	0.90 (0.71 to 1.13)	0.352
	Bacteraemia	329 (47.7%)	277 (40.1%)	84 (12.2%)			

*VPS28*, vacuolar protein sorting 28; *CPSF1*, cleavage and polyadenylation-specific factor 1; *ADCK5*, aarF domain-containing kinase 5; OR, odds ratio; CI, confidence interval; IPD, invasive pneumococcal disease. ^a^SNP positions listed are relative to the start of translation (in exon 5). ^b^Number of individuals (%). ^c^Comparison of heterozygotes [Aa] with homozygotes [AA + aa]. ^d^2 × 2 chi-squared comparison, one degree of freedom. ^e^Fisher's exact test (two-tailed). *P *values below 0.05 are highlighted in bold.

Two of the *NFKBIL2 *SNPs genotyped were found to be significantly associated with susceptibility to Gram-positive and pneumococcal bacteraemia in Kenyan children (rs4925858 and rs760477; Table 6). In each case the direction of association was of heterozygote protection, the same genetic model as that observed in the UK Caucasian study. Logistic regression analysis demonstrated no effect of age, comorbidity, HIV infection or gender on genotype. Comparison of ORs for rs4925858 and rs760477 did not demonstrate any evidence of heterogeneity between the UK and Kenyan case-control groups for either SNP. The strongest association was with rs760477; on combining and stratifying the UK and Kenyan study groups, heterozygosity at rs760477 was associated with significant protection against invasive bacterial disease overall (IPD in the UK study and overall bacteraemia in the Kenyan study; Mantel-Haenszel 2 × 2 χ^2 ^= 18.567, *P *= 1.6 × 10^-5^, OR = 0.66, 95% confidence interval for OR = 0.55 to 0.80) and against invasive pneumococcal disease specifically (Mantel-Haenszel 2 × 2 χ^2 ^= 11.797, *P *= 0.0006, OR = 0.67, 95% confidence interval for OR = 0.53 to 0.84). Heterozygosity at the neighbouring SNP rs4925858 was also found to be protective against both invasive bacterial disease overall (Mantel-Haenszel 2 × 2 χ^2 ^= 8.610, *P *= 0.003, OR = 0.76, 95% confidence interval for OR = 0.63 to 0.91) and IPD (Mantel-Haenszel 2 × 2 χ^2 ^= 9.104, *P *= 0.003, OR = 0.70, 95% confidence interval for OR = 0.55 to 0.88). None of the SNPs appeared to be associated with outcome of bacteraemia in these groups (data not shown), although the number of individuals in the poor outcome groups was small (mortality rates were 10% in the UK IPD study and 28% in the Kenyan study), resulting in a lack of power to examine possible effects of genotype on mortality.

**Table 6:** *NFKBIL2 *polymorphism genotype frequencies in Kenyan children with bacteraemia (overall, Gram-positive, and pneumococcal) and controls

Polymorphism/location (major/minor allele)	Status	Genotype distribution^a^			Total	Genotypic 3 × 2 chi-square (*P *value)	Heterozygote protection model^b^	
					
		AA	Aa	aa			OR (95% CI)	*P *value^c^
rs4925858, -3,771, 5' upstream (G/A)	Control	255 (46.4%)	247 (44.9%)	48 (8.7%)	550	4.104 (0.128)	0.80 (0.64 to 1.00)	0.053
	Bacteraemia	343 (49.9%)	271 (39.4%)	73 (10.6%)	687			
	Gram-positive bacteraemia	167 (49.9%)	125 (37.3%)	43 (12.8%)	335	6.806 (**0.034**)	0.73 (0.55 to 0.96)	**0.026**
	Pneumococcal bacteraemia	83 (48.8%)	62 (36.5%)	25 (14.7%)	170	6.900 (**0.032**)	0.70 (0.49 to 1.00)	0.052
rs760477, -263, intron 4 (C/T)	Control	262 (53.0%)	192 (38.9%)	40 (8.1%)	494	9.445 (**0.009**)	0.68 (0.53 to 0.87)	**0.002**
	Bacteraemia	403 (60.6%)	201 (30.2%)	61 (9.2%)	665			
	Gram-positive bacteraemia	197 (60.4%)	97 (29.8%)	32 (9.8%)	326	7.205 (**0.027**)	0.67 (0.49 to 0.90)	**0.007**
	Pneumococcal bacteraemia	93 (56.4%)	52 (31.5%)	20 (12.1%)	165	4.259 (0.119)	0.72 (0.50 to 1.05)	0.090

OR, odds ratio; CI, confidence interval. ^a^Number of individuals (%). ^b^Comparison of heterozygotes [Aa] with homozygotes [AA + aa]. ^c^2 × 2 chi-squared comparison, one degree of freedom. *P *values below 0.05 are highlighted in bold.

The SNP rs760477 is located within the fourth intron of *NFKBIL2*. The first seven exons of *NFKBIL2*, which surround rs760477, were then sequenced in 48 Kenyan individuals in an attempt to identify novel and potentially functional variants. The sequencing covered a 3,100 base pair region extending in a 3' direction from 780 base pairs prior to the start of transcription in exon 1. No novel exonic polymorphisms were identified with the exception of a synonymous polymorphism encoding asparagine at position 23 of the IκB-R protein, which has subsequently been listed on databases and named rs35913924. This SNP was then genotyped in the Kenyan cases and controls: the mutant allele was found to be uncommon (allele frequency 3.6%), and no association with disease was identified (3 × 2 χ^2 ^= 0.37, *P *= 0.83). The sequencing also confirmed the genotyping accuracy of rs760477 (100% concordance between direct sequencing and Sequenom genotyping).

## Discussion

In this study we demonstrate associations between common *NFKBIL2 *polymorphisms and susceptibility to IPD in UK Caucasian and Kenyan individuals. Important causes of false positive associations in genetic studies are a failure to adjust significance levels when multiple polymorphisms have been analysed, and confounding by population substructure. Nine polymorphisms were analysed, and applying a Bonferroni correction results in a threshold significance level of 0.0055, rather than 0.05. With this corrected significance level, rs760477, rs2306384 and rs4082353 in the UK population remain associated with protection against IPD. The Bonferroni correction, however, assumes that markers are independent, whereas many of the polymorphisms studied here are in strong or complete LD in UK individuals and are therefore not truly independent from each other. Applying instead a correction based on the total number of LD blocks and singleton (not part of a LD block) polymorphisms, five independent tests were performed in the UK study group, suggesting a threshold *P *value of 0.01 for statistical significance. The extent of LD was much less in Kenyan individuals, and as a result no LD blocks were predicted (Figure 2). In this setting, none of the polymorphisms in the Kenyan study reaches the Bonferroni-corrected *P *value threshold of 0.0055 to declare significance. Nevertheless, given the observed association between *NFKBIL2 *SNPs and IPD in UK individuals, the *a priori *probability that such a SNP protects against IPD in the Kenyan population might be expected to be higher than for a random marker, and in this situation the Bonferroni adjustment may be overly stringent. It is also noteworthy that the SNPs rs4925858 and rs760477 trend or associate in the same direction (heterozygote protection) in the Kenyan study as that observed in the UK study group, and combined analysis of the UK and Kenyan study groups using the Mantel-Haenszel test further strengthens the association between *NFKBIL2*rs760477 and IPD.

Addressing the possibility of population substructure, recent analysis of an extensive dataset of over 15,000 individuals from Britain demonstrated remarkably little evidence of geographic population differentiation within British Caucasians [18], and moreover our cases and controls are from a relatively restricted geographic area (Oxfordshire). Furthermore, the observation of a trend towards heterozygote protection against IPD in a second, independent study of African individuals provides additional support for an association between *NFKBIL2 *polymorphisms and pneumococcal disease. The results of the Kenyan study additionally suggest that the *NFKBIL2 *association may be with bacteraemia overall, rather than a specific effect on pneumococcal susceptibility, although this finding requires replication.

In general, a possible disadvantage for the use of the Kenyan samples as a replication study group is their different ethnic background: a lack of replication may reflect true ethnic heterogeneity in pneumococcal disease susceptibility. On the other hand, the study of a second population with differing patterns of LD may aid fine-mapping of associations within regions of strong LD, and it has been suggested that the demonstration of genetic associations with disease susceptibility across different populations is perhaps of even more value than the identification of population-specific effects [25]. The IPD-associated polymorphisms in the UK Caucasian study span a distance of 20 kb including the genes *NFKBIL2 *and *VPS28*. On the basis of these results alone it is not possible to further localise the disease association within this region, although the associations within the Kenyan study group appear to focus the association within *NFKBIL2*. Despite the use of such a transethnic mapping approach, the functional variant in *NFKBIL2 *that is responsible for the association with IPD remains unknown. Perhaps the most probable functional variant within *NFKBIL2 *is the coding change rs2306384, but it is noteworthy that this association did not replicate in the Kenyan study group. The SNP rs760477 is located within intron 4 and is unlikely itself to exert a functional effect, and no disease-associated polymorphisms were identified in the surrounding exons. The polymorphism rs4925858 is located 1,650 base pairs before the transcription start, and could conceivably affect a regulatory region such as a promoter, enhancer or silencer.

The mechanism by which IκB-R variation influences susceptibility to IPD is also unclear. One possibility is through an effect on CCL5 expression, which has been reported to be upregulated in lung epithelial cells following overexpression of IκB-R [16]. The mechanism behind this cytokine-induced upregulation appears to be sequestering of transcriptionally repressive NF-κB p50 homodimer subunits by IκB-R, thereby facilitating NF-κB-mediated gene transcription of CCL5 [16]. Both CCL5 mRNA and protein expression are stimulated following exposure to the pneumococcal proteins pneumolysin and choline-binding protein A in dendritic cells, and furthermore CCL5 blockade during pneumococcal carriage in mice is associated with an attenuated immune response and greater transition to lethal pneumonia [26,27]. Further research is required to examine the possible role of IκB-R in regulation of CCL5 during pneumococcal disease, and indeed to identify the cellular roles of IκB-R more generally. This protein has been relatively neglected compared with the extensive literature on other IκBs, and it remains unclear for example which specific NF-κB dimers interact with IκB-R [14,16].

The direction of association with disease is noteworthy: heterozygosity was associated with protection against IPD in each study population. The finding of heterozygote protection is unusual in genetic disease association studies, but is well described in the study of human infectious disease genetic susceptibility - examples include sickle cell trait and malaria, prion protein gene variation and spongiform encephalopathy, and human leukocyte antigen and HIV/AIDS disease progression [28-30]. More recently, heterozygosity at loci within both the Toll-like receptor adaptor protein Mal/TIRAP and *NFKBIZ *have been found to associate with protection against IPD [10,31]. Interestingly, studies in animal populations have found that increased levels of genome-wide heterozygosity correlate with overall fitness, and more specifically with resistance to infectious disease; for example, resistance to bovine tuberculosis in the Iberian wild boar [32]. These animal studies raise the possibility that heterozygote advantage against infectious disease may be a more widespread phenomenon in humans than previously considered. The biological mechanisms that underlie this remain unclear, although in the setting of inflammatory signalling pathways it has been speculated that homozygote states may lead to extremes of inflammatory response that, under certain circumstances, are detrimental to the host, whereas heterozygosity may result in intermediate signalling that leads to an optimal inflammatory response [31]. Such a model will be further modified by environmental exposures, and differing burdens of bacterial disease may in part account for the observed variation in *NFKBIL2 *allele frequencies between European and African populations.

Finally, it is interesting that four out of the five IκB genes studied to date show apparent associations with susceptibility to IPD [9,10]. This further highlights the importance of the control of NF-κB in the host immune response, and suggests that the remaining members of the IκB family are likely to be promising candidates for a role in pneumococcal susceptibility. Study of the genetic basis of NF-κB inhibition may be increasingly relevant given current interest in the regulation of NF-κB activity as a therapeutic target for inflammatory disease [33]. Within the field of infectious disease, inhibition of NF-κB has been demonstrated to improve outcome in animal models of sepsis and pneumococcal meningitis [34,35]. The anti-inflammatory activity of glucocorticoids is mediated at least in part through physical interference of the glucocorticoid receptor complex with NF-κB DNA binding and increased synthesis of IκB [36], and there is some evidence of benefit from corticosteroids in the treatment of pneumococcal meningitis and perhaps also severe community-acquired pneumonia [37,38]. Taken together, these findings raise the intriguing possibility that anti-inflammatory treatments such as glucocorticoids may be more effective if tailored on the basis of an individual's genetic profile of NF-κB activation.

## Conclusions

Our study demonstrates associations between common *NFKBIL2 *polymorphisms and susceptibility to IPD in European and African populations. These findings further support a central role for regulation of NF-κB in human host defence against pneumococcal disease.

## Key messages

•	Common polymorphisms in the gene *NFKBIL2 *associate with susceptibility to IPD in European and African populations.

•	The parallel study of disease phenotypes in European and African populations (a trans-ethnic mapping approach) facilitates fine-mapping of genetic associations within regions of strong LD.

•	Genetic variation in control of the proinflammatory transcription factor NF-κB appears to play a key role in host defence against pneumococcal disease.

## Abbreviations

CCL5: C-C motif ligand 5; IκB: inhibitor of NF-κB; IL: interleukin; IPD: invasive pneumococcal disease; LD: linkage disequilibrium; NF: nuclear factor; OR: odds ratio; PCR: polymerase chain reaction; RANTES: 'regulated upon activation normal T cell expressed and secreted'; SNP: single nucleotide polymorphism; TNF: tumour necrosis factor.

## Competing interests

The authors declare that they have no competing interests.

## Authors' contributions

SJC, CCK and FOV performed genotyping and statistical analysis. SJC drafted the manuscript. SS, CEM, RJOD, NPD, NP, DWC, JAB, TNW and JAS enrolled patients, collected samples and data, and defined phenotypes. DWC, JAS and AVSH coordinated the study. SJC, CCK, FOV, AR, AW, DWC, JAB, TNW, JAS and AVSH contributed to the conception and design of the project. All authors read and approved the final manuscript.
